# Prevalence and Incidence of Syphilis among Volunteer Blood Donors in Israel

**DOI:** 10.1155/2014/154048

**Published:** 2014-04-22

**Authors:** Leibovici Vera, Donchin Milka, Strauss-Liviatan Nurith, Shinar Eilat

**Affiliations:** ^1^Department of Dermatology, Hadassah-Hebrew University Hospital, P.O. Box 12018, Jersalem, Israel; ^2^Brown School of Public Health, Hadassah University Hospital, Jersalem, Israel; ^3^Private Practice, P.O. Box 84027, 90805 Mevaseret Zion, Israel; ^4^Magen David Adom National Blood Services, 52621 Ramat Gan, Israel

## Abstract

Data of 1,290,222 volunteer blood donors, in a 5-year period, was analyzed for prevalence and incidence of syphilis. Subsequent testing of donations positive in* Treponema pallidum* hemagglutination assay included Venereal Disease Research Laboratory and fluorescent Treponemal antibody absorption. Stepwise logistic regression model was used to identify positive syphilis serology. Prevalence of syphilis was 47 : 100,000, similar in men and women and increased significantly with age (*P* < 0.001). Native Israelis had the lowest prevalence rate of syphilis (21 : 100,000), while a significantly higher prevalence was found among immigrants from Africa, Eastern Europe, and South America (odds ratios of 19.0, 10.8, and 7.3, resp., *P* < 0.001 for each). About 33.2% of the seropositive donors had evidence of recent infection, and 66.8% had past infections. Incidence rate reached 8 : 100,000 person-years. Coinfection with HIV, HCV, and HBV was calculated as 8%, 1.88%, and 0.37% for positive donations, respectively. The data support the need to continue screening blood donors in Israel for syphilis and employ preventive measures to populations at risk, in order to improve public health, blood safety, and quality. A subsequent study to assess blood donors' knowledge, attitude, and behavior is planned. In times of global migration this information may be useful to blood services worldwide.

## 1. Introduction 

Syphilis is a sexually transmitted disease (STD) caused by* Treponema pallidum*, which can also be transmitted via accidental direct inoculation, transplacenta during pregnancy, and, rarely, via blood transfusion [1, 2].

Although the value of routine serologic screening of blood donors for syphilis has been a question in debate for years [3–5], and refrigerated blood components are less infective for syphilis, transmissions through blood components still occur [6]. Therefore, standard operating procedures of blood establishments worldwide include demands/recommendations for such screening [7, 8]. New draft guidance for screening, testing, and management of blood donors and components was recently distributed by the FDA [9].

In many parts of the world, the incidence and prevalence of syphilis still remain high in both volunteer and family/replacement blood donors [10–15]. There are numerous reports in high-risk groups in the literature, both from developed and developing countries, indicating rising prevalence and incidence of syphilis [16–20].

In Israel, screening tests for syphilis are conducted in community STD clinics for the general and high-risk populations, by the National Blood Services on each blood unit donated (all collected from volunteer blood donors), and also in all pregnant women. Confirmatory tests for all positive samples are performed exclusively by the Central National Reference Laboratory for Venereal Diseases of the Ministry of Health (MOH), thus forming a national registry for surveillance and followup.

Data reported by the Israeli MOH to the WHO regional office in Europe showed that the incidence of syphilis in the general population in Israel was 2.52, 5.8, and a low 0.7 per 100,000 in 1996, 2001, and 2005, respectively [21]. Although the incidence of syphilis in Israel in the general population in 2005 was rather low, further reports between 2004 and 2009 in people with high-risk sexual behavior show an increase of the prevalence and incidence of syphilis in this specific group [22–25]. We studied the prevalence and incidence of syphilis and coinfection with other transfusion-transmitted diseases in volunteer blood donors of the Israeli National Magen David Adom Blood Services (MDABS) during 2005–2009 to assess the determinants of this sexually transmitted disease in this selected population.

## 2. Materials and Methods

### 2.1. Study Population

All blood donors in Israel are nonremunerated volunteers. Units are collected throughout the country in mobile vehicles or in fixed-site donor rooms. Before donation each potential donor fills a detailed health history questionnaire, which includes data regarding age, gender, country of birth, and year of immigration (if applicable), and questions concerning the donor's general health, lifestyle and risk behavior, followed by a short private interview, blood pressure and hemoglobin determination, and physical examination. Donors may be disqualified and deferred from donating based on their written and verbal answers or on previous information in the MDABS database. Relevant deferral criteria include, among others, permanent deferral for known HIV or hepatitis, by past testing or self-report, men who have had sex with men (MSM) since 1977, and people who have been given money for sex (sex workers). A 12-month temporary deferral includes donors who were treated for syphilis or gonorrhea within the previous year and those who had sex with people mentioned above [7]. The study was carried out using the computerized database of MDA Blood Services donors' records for the period 2005–2009.

### 2.2. Laboratory Tests

#### 2.2.1. Screening for Syphilis (see Figure 1)

Tests were done using a specific serological* Treponema Pallidum *hemagglutination assay (TPHA) of Biokit-Syphagen TPHA autoindirect hemagglutination test (Biokit, Barcelona, Spain), performed on Olympus PK 7200 blood-typing equipment (Olympus, Japan). Each initial reactive sample was further tested in duplicate, according to the manufacturer's instructions. If two out of three results were positive (Repeat Reactive), the unit was discarded and the sample was sent to the National Reference Laboratory for Venereal Diseases of the Israeli Ministry of Health for serological confirmation testing using a different TPHA hemagglutination assay, the nonspecific treponemal assay Venereal Disease Research Laboratory (VDRL), using BD VDRL Antigen (Becton Dickinson, USA), and fluorescent Treponemal absorption test (FTA-ABS) with Trepo-Spot IF, Fluoline H (Biomerieux France).

According to the confirmatory results donors were defined as

- “Recent/Active Infection”: if confirmatory results were one of the following: VDRL+ TPHA+ FTA+; VDRL− TPHA− FTA+; VDRL+ TPHA− FTA+ or VDRL+ TPHA+ FTA−, the donors have not been treated and therefore may be infectious [26];
- “Past Infection/Serological Scar”: if confirmatory results were VDRL− TPHA+ FTA+

The test was defined as false positive when VDRL− TPHA+ and FTA−.

*Prevalence* was defined as the number of TPHA positive donors in the total donors' population.* Incidence* was defined as the number of new TPHA positive donors in the total donors' population.* Seroconversion *was defined when the current donation from a previously seronegative repeat donor gave a seropositive confirmed reaction.

#### 2.2.2. Screening for Other Transfusion Transmitted Viruses

Tests were performed at MDABS laboratories using chemiluminescentimmunoassay for human immunodeficiency virus (HIV), hepatitis B (HBV), hepatitis C (HCV), and human T-lymphotropic virus (HTLV) (Prism, Abbott laboratories, USA), and Individual Unit Nucleic Acid Testing (ID-NAT) for HIV, HBV, and HCV using Procleix Ultrio (Novartis, USA).

### 2.3. Statistical Analyses

Seroprevalence was defined as the proportion of donors serologically confirmed as positives from the total number of donors and was given with 95% confidence intervals.

The seroconversion rate per 100,000 person-years was calculated by the number of donors who seroconverted, divided by the total number of person-years between consecutive donations, multiplied by 100,000.

Proportion comparisons were performed with the normal approximation. The chi-square test was used to test the association between categorical variables.

Two models of stepwise logistic regression were used to investigate which variables are associated with total seropositivity (Yes/No) and with recent infection (Yes/No). We estimated the models with the following explanatory variables: age, country of birth (grouped into Israeli-born, East Europe, and former USSR countries, West Europe, North America, and Oceania, Africa, Asia, and South America), year of immigration (equals 1 for immigration year ≥ 1990 and 0 before 1990), and the 2-way interactions between immigration year and country group. Country group was a categorical variable, with Israel as the reference category to each of the other five country groups. We used the forward conditional method for variable selection. The final models are presented. *P* values are given for the significant variables, and estimates of the odds ratios with their corresponding 95% confidence intervals are given.

## 3. Results

Data for analysis included a total of 1,290,222 valid blood donations, collected from 605,549 donors, with an average of 2.1 units/donor (range 1–57). About 69% of the donors were men and 31% were women. Median age was 29, ranging from 17 to 70 years. Mean age of the donors was 33 ± 12.4 years. About 79% of the blood donors were born in Israel, and 21% had emigrated from different countries (Table 1).

Out of the 605,549 volunteers who donated blood in 2005–2009, 283 were seropositive, indicating a prevalence of 47 per 100,000. Prevalence rates of all the syphilis seropositive donors (with both recent and past infection) analyzed by gender, age, country of origin, and time of immigration to Israel are depicted in Table 1.

Men and women had similar prevalence rates of syphilis of 45 and 49 per 100,000, respectively. There was a significant increase (*P* < 0.001) in the prevalence rate of syphilis with age: donors age 45 years and older, who donated about one-fifth of the total number of units, comprised 36.7% of the seropositives. Prevalence rates were 7.4, 6.5, and 3.7 times higher in donors age 45 or older, 35–44, and 25–35, respectively, compared to ≤24 years.

When analyzed by country of origin, the highest rates of seropositivity were detected in donors born in Eastern Europe and Africa (192 per 100,000 and 178 per 100,000, resp.), when compared to native Israeli.

Ninety-four of the 283 seropositive donors (33%) were diagnosed as Recent Infections by the National Reference Laboratory for Venereal Diseases, and 189/283 (67%) were defined as Past Infection/Serological Scars. The distribution of seropositives between donors defined as recent or past infections was similar between the two groups (Table 2) including following a review of the donors' history and questionnaires.

The logistic regression model (Table 3) enables identification of the marginal contribution of the above determinants to the risk of seropositivity, both total and recent infection.

Age increased the risk of syphilis in the given population of blood donors (controlling for the other variables in the model). Every additional year of age adds 6% to the risk of either total seropositivity or recent infection (odds ratio = 1.06, *P* < 0.001). The interaction of immigration year (above and below 1990) and country of birth group was also significant. More specifically, new immigrants (immigration year ≥ 1990) from Africa, Eastern Europe, and South America had a significantly higher risk for syphilis (with odds ratios of 19.0, 10.8, and 7.3, resp., *P* < 0.001 for each) compared to the risk of Israeli-born donors and those who immigrated before 1990. Similar results are presented for the recent infection model.

Seroconversion was found in 51 donors, giving an incidence rate of 8 per 100,000 person-years. No significant differences were found when compared to the other seropositive donors, by the studied parameters.

**Figure 2:**
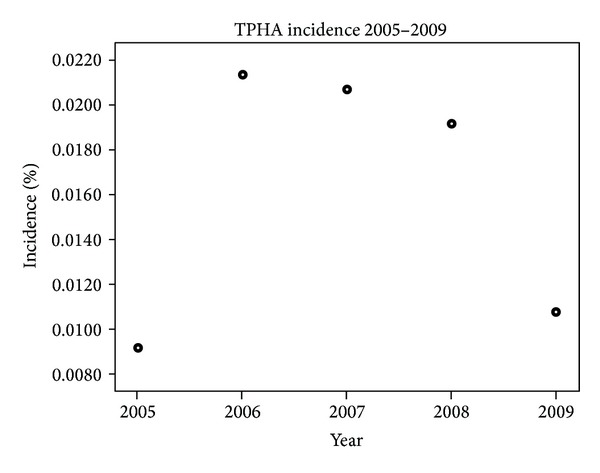
Incidence of TPHA seropositivity during the study period.

Incidence by year is given in Figure 2.

Coinfection of seropositivity for syphilis with other transfusion-transmitted diseases was found: two out of 25 HIV-positive donors were seropositive for syphilis (8%), compared to 0.046% among the HIV-negative blood donors (*P* < 0.001). In addition, 0.37% and 1.88% of the seropositive donors for hepatitis B and C, respectively, were positive for syphilis, compared with 0.046 and 0.045% among the negative donors for these two hepatitis viruses (*P* < 0.001 for each test).

## 4. Discussion

Previous studies of the prevalence of syphilis in Israel mostly took place among people with high-risk sexual behavior, such as MSM or sex workers. Only in one study, reported in 2005 and conducted in a limited region, a low incidence of 0.7 cases of syphilis in 100,000 people was found [21].

The volunteer blood donors are considered a selective population, since they are prescreened for previous diseases and sexual behavior, both by the detailed questionnaire and during a discrete personal interview before donation. However, since blood donation is performed by all citizens, it could provide an updated picture about incidence and prevalence of syphilis in this population.

Data obtained during the period 2005–2009 show a prevalence of 47 per 100,000, of which 33% were diagnosed as recently infected and 67% were defined as past infection/“serological scars.” In addition, an incidence of 8 per 100,000 person-years was detected, which is an 11.4-fold increase since 2005.

Three major variables in our study had statistically significant impact on the risk of seropositivity (either for the entire group or for recent infection): age of the donors, their country of origin, and the time of immigration to Israel.

*Age.* The prevalence rates of syphilis increased with age. 

*Donors*. The 35–44 year age group and those older than 45 years had a 6.5-fold and 7.4-fold higher prevalence of syphilis, respectively, when compared to the younger donors (aged 24 years or less). This is in agreement with others with similar findings among blood donors (18) or who described an increase of primary and secondary syphilis in older adults in the USA [27, 28]. 

*Country of Origin and Time of Immigration*. Israel is a country that absorbs Jewish immigrants from all over the world. In particular, from 1990 to 2001 more than 900,000 immigrants came to the country (13% of the total Israeli population), mostly from the former Soviet Union and from Africa [29].

Our study revealed that the new immigrants from Africa and from Eastern Europe who arrived in Israel after 1990 had a 19-fold and 10.8-fold (resp.) higher risk for syphilis seropositivity than donors born in Israel. The findings were similar to the prevalence in their countries of origin, as reported by the Israeli MOH [30] and the 2001 WHO European Health database [21]. These results are in accordance with the high incidence of syphilis in the former communist countries of Eastern Europe, with rates of 262, 245, and 150 cases per 100,000 in the Russian Federation, Kazakhstan and Ukraine, respectively [21], and with data published from Africa in 2001, with infection rates of 1000, 300–400, and 100–200 cases per 100,000 found in Zambia, Kenya, and Benin, respectively [31].

The study also depicted an association between syphilis and other transfusion-transmitted diseases such as HIV, hepatitis B, and C, when compared to the donor population that was seronegative for syphilis, and it concurred with our previous observations of higher prevalence of hepatitis C among blood donors who immigrated to Israel from the former USSR [32].

It is worth mentioning that about 62% of the units were donated by native Israelis, with the lowest risk for recent infection, thus contributing to the safety of the national inventory.

In view of the ongoing discussion regarding the recent FDA's draft Recommendation for Screening, Testing and Management of Blood Donors, and Blood Components Based on Screening Tests for Syphilis [9], our findings demonstrate that screening for syphilis may still retain certain value in Israel and should be considered in some other regions in the world, depending on their blood donors' epidemiology data.

Given the high prevalence of syphilis in Israel allied with active immigration, special attention should be invested in targeting preventive measures to the blood donor populations and populations at risk. A future study is planned to assess the knowledge, attitude, and behavior of the volunteer blood donors' population. These measures will most probably improve public health and increase blood safety and quality.

In times of global migration the reported information may be useful to blood services worldwide.

## Figures and Tables

**Figure 1:**
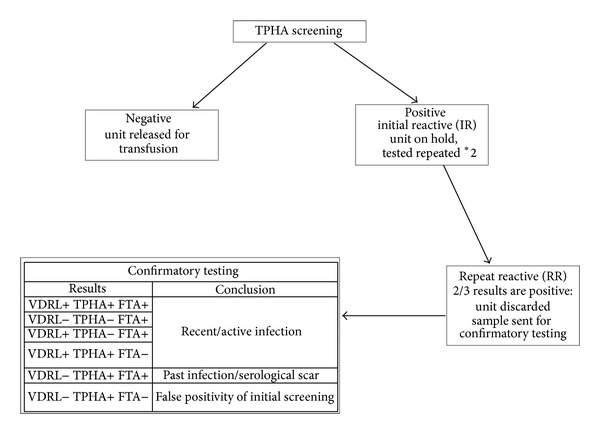
Algorithm for syphilis screening and confirmatory testing.

**Table 1:** Prevalence (%) of seropositive donors by gender, age, country of birth, and year of immigration.

Variable	Number of donations	Number of donors	Number of seropositives	Prevalence (%)	95% C.I. of prevalence
Total	1,290,222	606,549	283	0.0467	(0.0415, 0.0524)
Gender					
Men	945,653	417,475	189	0.0453	(0.0393, 0.0522)
Women	344,569	189,074	94	0.0497	(0.0406, 0.0608)
Age					
≤24	440,712	207,203	24	0.0116	(0.0078, 0.0172)
25–34	357,888	170,781	74	0.0433	0.0345, 0.0544)
35–44	217,965	107,251	81	0.0755	(0.0608, 0.0938)
≥45	273,657	121,314	104	0.0857	(0.0708, 0.1039)
Country of origin					
Israel	1,022,071	474,140	101	0.0213	(0.0175, 0.0259)
Asia	29,423	14,580	17	0.1166	(0.0728, 0.1867)
Africa	9,921	5,047	9	0.1783	(0.0938, 0.3386)
USSR and Eastern Europe	141,894	70,766	136	0.1922	(0.1625, 0.2273)
Western Europe North America and Oceania	66,106	32,370	11	0.0340	(0.0190, 0.0608)
South America	19,159	8,863	9	0.1015	(0.0534, 0.1929)
Unknown	1,648	783	0		
Time of immigration					
Israeli born	1,022,071	474,140	101	0.0213	(0.0175, 0.0259)
<1990	109,483	47,774	39	0.0816	0.0597, 0.1116)
≥1990	142,292	72,188	136	0.1884	(0.1593, 0.2228)
Unknown	16,376	12,447	7	0.0562	(0.0272, 0.1161)

**Table 2:** Distribution of donors with recent versus past syphilis by country of origin, age, and time of immigration.

	Active infection	Past infection	Total
Country of origin			
** Total**	**94 (100%)**	**189 (100%)**	**283 (100%)**
** **Israel	36 (38.3%)	65 (34.4%)	**101 (35.7%)**
** **Asia	8 (8.5%)	9 (4.8%)	**17 (6.0%)**
** **Africa	4 (4.3%)	5 (2.6%)	**9 (3.2%)**
** **USSR and Eastern Europe	39 (41.5%)	97 (51.3%)	**136 (48.1%)**
** **Western Europe North America and Oceania	4 (4.3%)	7 (3.7%)	**11 (3.9%)**
** **South America	3 (3.2%)	6 (3.2%)	**9 (3.2%)**
Age (years)			
≤24	9 (9.6%)	15 (7.9%)	**24 (8.5%)**
25–34	23 (24.5%)	51 (27.0%)	**74 (26.1%)**
35–44	28 (29.8%)	53 (28.0%)	**81 (28.6%)**
≥45	34 (36.2%)	70 (37.0%)	**104 (36.7%)**
Year of immigration			
<1990	15 (16.1%)	24 (13.1%)	**39 (14.1%)**
≥1990	42 (45.2%)	94 (51.4%)	**136 (49.3%)**
Israeli born	36 (38.7% )	65 (35.5%)	**101 (36.6%)**

**Table 3:** Odds ratio (OR) of the determinants of seropositivity for syphilis, total and recent infection—logistic regression models.

Variable	Total seropositivity						Recent infection					
	*B*-Coefficient in model	Standard error	Sig.	OR	95% C.I. for OR		*B*-Coefficient in model	Standard error	Sig.	OR	95% C.I. for OR	
					Lower	Upper					Lower	Upper
Age	0.055	0.004	<0.0001	1.06	1.047	1.065	0.054	0.008	<0.0001	1.06	1.040	1.072
Immigration Year 1990* Country Group			<0.0001						<0.0001			
Imm. Year 1990 by Country Group (Asia)	1.286	1.004	0.2004	3.62	0.505	25.906	2.295	1.011	0.0231	9.92	1.368	71.928
Imm. Year 1990 by Country Group (Africa)	2.944	0.419	<0.0001	19.00	8.360	43.168	3.256	0.596	<0.0001	25.94	8.068	83.412
Imm. Year 1990 by Country Group (Eastern Europe)	2.381	0.127	<0.0001	10.82	8.442	13.870	2.101	0.226	<0.0001	8.17	5.244	12.731
Imm. Year 1990 by Country Group (Western Europe)	−0.711	1.004	0.4789	0.49	0.069	3.514	0.297	1.011	0.7687	1.35	0.186	9.757
Imm. Year 1990 by Country Group (South America)	1.991	0.456	<0.0001	7.32	2.994	17.912	2.487	0.596	<0.0001	12.02	3.738	38.677
